# 365. Durable efficacy and robust CD4+ T-cell count improvement observed across age, race, sex, and geographic subgroups of heavily treatment-experienced people with multidrug-resistant HIV-1 after 240 weeks of fostemsavir treatment

**DOI:** 10.1093/ofid/ofad500.435

**Published:** 2023-11-27

**Authors:** Alftan Dyson, Judith A Aberg, Jean-Michel Molina, Isabel Cassetti, Michael Kozal, Sandra Treviño-Pérez, Gulam Latiff, Jacob Lalezari, Gilles Pialoux, Bo Li, Manyu Prakash, Andrew Clark, Allan R Tenorio, Amy Pierce, Max Lataillade

**Affiliations:** ViiV Healthcare, Durham, North Carolina; Icahn School of Medicine at Mount Sinai, New York, New York; UNiversity of Paris CIte, Paris, Ile-de-France, France; Helios Salud, Capital Federal, Argentina; Stanford University, Stanford, California; Mexico Centre for Clinical Research, Mexico City, Estado de México, Mexico; Maxwell Centre, Durban, KwaZulu-Natal, South Africa; Quest Clinical Research, San Francisco, California; Hôpital Tenon, Assistance Publique-Hôpitaux de Paris, Paris, Ile-de-France, France; GSK, Collegeville, Pennsylvania; ViiV Healthcare, Brentford, UK, Brentford, England, United Kingdom; ViiV Healthcare, Brentford, UK, Brentford, England, United Kingdom; ViiV Healthcare, Durham, North Carolina; ViiV Healthcare, Durham, North Carolina; ViiV Healthcare, Durham, North Carolina

## Abstract

**Background:**

Fostemsavir (FTR), the prodrug of the first-in-class attachment inhibitor temsavir, is indicated in combination with other antiretrovirals (ARVs) for heavily-treatment experienced (HTE) people with multidrug-resistant HIV-1 unable to construct suppressive regimens. After ∼5 years in the phase 3 BRIGHTE study, overall virologic response rate (HIV-1 RNA < 40 c/mL, Snapshot) with FTR + optimized background therapy (OBT) was 45% (120/267) in the Randomized Cohort (RC) and 22% (20/92) in the Non-Randomized Cohort (NRC). We present long-term efficacy of FTR + OBT among subgroups in BRIGHTE.

**Methods:**

BRIGHTE included HTE adults with HIV-1 (N=371) failing their current ARV regimen (HIV-1 RNA > 400 c/mL) with ≤ 2 fully active and approved ARVs remaining. Participants with 1 or 2 ARVs entered the RC and received open-label FTR + OBT after an 8-day blinded placebo-controlled period; those with 0 ARVs entered the NRC and received open-label FTR + OBT from Day 0. Virologic and immunologic responses were analyzed by baseline (BL) demographics and disease characteristics.

**Results:**

Of 371 participants (RC, n=272; NRC, n=99), 290 (78%) were male, 166 (45%) were aged ≥ 50 years, and 259 (70%) were White. Virologic response rates were generally comparable among subgroups and consistent with overall response rate (Table 1). Similar efficacy was observed in RC participants with 1 or 2 fully active ARVs in OBT, while both RC and NRC participants with high BL viral loads or low BL CD4+ T-cell counts had lower response rates. Nearly all subgroups demonstrated robust and continuous increases in CD4+ T-cell count from Weeks 96 to 240, with mean change from BL exceeding 200 cells/mm^3^ in all RC subgroups, including those with BL viral loads ≥ 100,000 c/mL or CD4+ T-cell counts < 20 cells/mm^3^ (Table 2).

**Figure:**
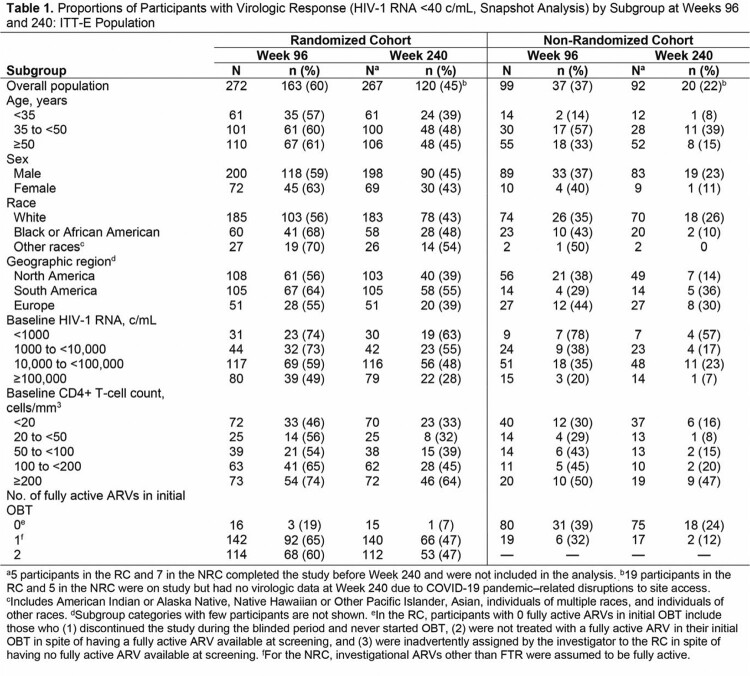

**Figure:**
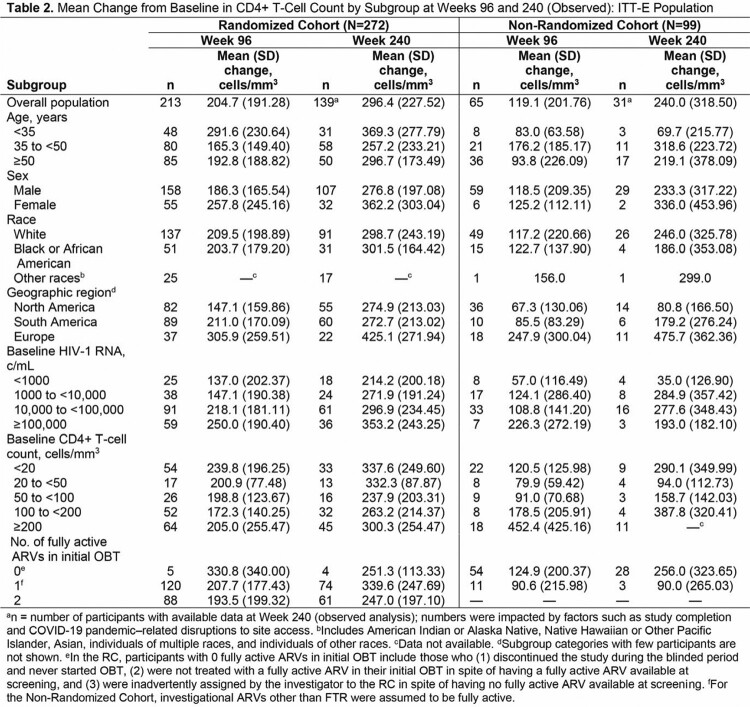

**Conclusion:**

Virologic response with FTR + OBT in HTE adults with advanced HIV-1 and limited treatment options was durable over ∼5 years, with no major differences among subgroups based on age, sex, race, or geographic region. Robust and continuous CD4+ T-cell increases were also observed, with numerically greater improvements in subgroups with lowest BL CD4+ T-cell counts. These data highlight the role of FTR as a treatment option for HTE people with multidrug-resistant HIV-1 regardless of demographic or disease characteristics.

**Disclosures:**

**Alftan Dyson, PharmD**, GSK: Stocks/Bonds|ViiV Healthcare: Employment **Judith A. Aberg, MD**, Emergent BioSolutions: Institutional research grants|Frontier Technologies: Institutional research grants|Gilead: Institutional research grants|GSK: Advisor/Consultant|GSK: Institutional research grants|Janssen: Institutional research grants|Merck: Advisor/Consultant|Merck: Institutional research grants|Pfizer: Institutional research grants|Regeneron: Institutional research grants|ViiV Healthcare: Advisor/Consultant|ViiV Healthcare: Institutional research grants **Jean-Michel Molina, MD; PhD**, Aelix: Advisor/Consultant|Gilead: Advisor/Consultant|Merck: Advisor/Consultant|ViiV Healthcare: Advisor/Consultant **Isabel Cassetti, MD**, Gilead: Advisor/Consultant|Gilead: Institutional research grants|GSK: Advisor/Consultant|Janssen: Institutional research grants|Merck: Advisor/Consultant|Merck: Institutional research grants|ViiV Healthcare: Institutional research grants **Michael Kozal, MD**, ViiV Healthcare: Grant/Research Support|ViiV Healthcare: Royalties/Licenses for chapters authored for UpToDate **Bo Li, PhD**, GSK: Employment|GSK: Stocks/Bonds **Manyu Prakash, PhD**, GSK: Stocks/Bonds|ViiV Healthcare: Employee **Andrew Clark, MD**, GSK: Stocks/Bonds|ViiV Healthcare: Employee **Allan R. Tenorio, MD**, GSK: Stocks/Bonds|ViiV Healthcare: Employee **Amy Pierce, BS**, GSK: Stocks/Bonds|ViiV Healthcare: Employee **Max Lataillade, DO**, GSK: Stocks/Bonds|ViiV Healthcare: Employee

